# Development of a Competition-Binding Assay to Determine Binding Affinity of Molecules to Neuromelanin via Fluorescence Spectroscopy

**DOI:** 10.3390/biom9050175

**Published:** 2019-05-08

**Authors:** Jackson Fink, Heather Pathak, John Smith, Cindy Achat-Mendes, Robert L. Haining

**Affiliations:** School of Science and Technology, Georgia Gwinnett College, 1000 University Center Lane, Lawrenceville, GA 30043, USA; jfink.fl@gmail.com (J.F.); heatherpathak@gmail.com (H.P.); commonman78@hotmail.com (J.S.); cachatme@ggc.edu (C.A.-M.)

**Keywords:** dopamine, neuromelanin (NM), binding constant (Kd), nicotine, fluorescence, Parkinson’s

## Abstract

Neuromelanin, the polymeric form of dopamine which accumulates in aging neuronal tissue, is increasingly recognized as a functional and critical component of a healthy and active adult human brain. Notorious in plant and insect literature for their ability to bind and retain amines for long periods of time, catecholamine polymers known colloquially as ‘melanins’ are nevertheless curiously absent from most textbooks regarding biochemistry, neuroscience, and evolution. Recent research has brought attention to the brain pigment due to its possible role in neurodegeneration. This linkage is best illustrated by Parkinson’s disease, which is characterized by the loss of pigmented dopaminergic neurons and the ‘white brain’ pathological state. As such, the ability to determine the binding affinity of neurotoxic agents, as well as any potential specific endogenous ligands to neuromelanin are of interest and potential value. Neuromelanin has been shown to have saturable binding interactions with nicotine as monitored by a fluorimeter. This interaction provides a signal to allow for a competition-binding assay with target molecules which do not themselves produce signal. The current report establishes the viability of this competition assay toward three compounds with central relevance to Parkinson’s disease. The K_d_ of binding toward neuromelanin by methyl-phenyl-pyridinium ion (MPP+), dopamine, and 6-hydroxydopamine were found to be 1 mM, 0.05 mM, and 0.1 mM, respectively in the current study. In addition, we demonstrate that 6-hydroxydopamine polymerizes to form neuromelanin granules in cultured dopaminergic neurons that treated with 2,4,5-trihydroxy-l-phenylalanine. Immunohistochemical analysis using fluor-tagged anti-dopamine antibodies suggests that the incorporation of 6-hydroxydopamine (following internalization and decarboxylation analogous to levodopa and dopamine) alters the localized distribution of bound dopamine in these cells.

## 1. Introduction

### 1.1. Neuromelanin and Parkinson’s Disease

Neuromelanin (NM) is a pigment consisting of conjugated 5,6 dihydroxyindole rings resulting mainly from the spontaneous co-polymerization of dopamine and cysteine into a granule along with an apparent set of critical lipids and peptides that is normally found to accumulate in human brain tissue as a normal part of the aging process [1]. It is believed to arise as a result of unusually high dopamine concentrations which accumulate temporarily in the cytoplasm of dopaminergic neurons following repeating localized firing patterns and subsequent uptake through dopamine transporters (DATs), prior to sequestration into dopaminergic vesicles through vesicular monoamine transporter (VMAT) activity [2]. The more stable, electrically reduced catecholamine state of dopamine is normally in a steady-state/equilibrium with the oxidized quinone form through the action of tyrosinase [3]. Adrenochrome spontaneously initiates the polymerization process in concentrated solutions and is the source of much of the oxidative stress in cells which harbor dopamine. Above a certain catecholamine threshold, the thiol antioxidant buffering defense mechanisms, consisting of glutathione and cysteine in redox equilibrium with their corresponding disulfide moieties, are overwhelmed, raising the oxidation state and allowing dopamine to proceed to the quinone. In doing so, a spontaneous cascade of events is initiated which leads to polymer formation and the eventual appearance of neuromelanin granules [4,5].

Considered an aging pigment, the granules resulting when the melanin polymer is carefully packaged into double membrane-bound structures that are found tightly associated with an assortment of characteristic lipids and proteins [6,7]. It is highly concentrated in areas including the substantia nigra, a region most associated with the initiation of dopaminergic transmission to enable muscle movement. The profound loss of neuromelanin during the latter stages of Parkinson’s disease made this disorder known as the disease of the white brain over 300 years ago, yet we still know very little about its function [8]. Iron is found bound in a highly specific manner to the synthetic and natural neuromelanin polymer [9,10,11] and some have since suggested that NM may act in a cytoprotective role against rogue metal ions or neurotoxins [12,13,14]. Famously, the chemical 1-Methyl-4-phenyl-1,2,3,6-tetrahydropyridine (MPTP) was been shown to induce Parkinson’s disease in humans and animal models after conversion to the pyridinium ion (MPP+), uptake through cation transporters, and subsequent binding to and accumulation by the NM granules [15,16,17]. In other instances, agents may disrupt the putative function of NM by incorporating into the polymer itself prior to sequestration, a situation we hypothesize to be the case with 6-hydroxydopamine. That is, given that NM is a polymer of dopamine and cysteine, the structural and chemical similarity with 6-hydroxydopamine makes incorporation highly likely. The accumulation of pigment over time is seen then, in this view, as an attempt to overcome the inevitable effects of toxic insult and aging. Parkinson’s disease is often therefore described by the death of dopaminergic neurons in the substantia nigra of the brain, and neurons with high concentrations of NM are more susceptible to death in Parkinson’s disease [16,17,18].

### 1.2. Role of Nicotine in Protection from PD-Inducing Agents

Ongoing research in our laboratory is focused on attempts to understand the nature of chemical toxin attraction to NM granules and to establish a ranking based on equilibrium binding constants. Our umbrella hypothesis is that neuromelanin granules themselves have a critical function in human brain that is not well understood, and that many Parkinsonian-inducing agents owe their distinct toxicity to disruption of such normal functioning. In some cases, dysfunction and subsequent neuronal death may result from the attraction of toxic molecules to and concentration in cells containing the unique environment afforded by the iron-bound pigmented granule, like that observed from exposure to MPTP. At the opposite end, certain compounds have been shown to have a protective effect against idiopathic as well as chemically-induced Parkinson’s disease, of primary current interest among them being nicotine [19,20,21].

Nicotine has been shown to have neuroprotective effects in animal models of PD when applied to *substantia nigra* cells prior to exposure to MPP+ [22]. Epidemiological studies confirm that tobacco smokers and other nicotine users have lower incidences of idiopathic PD. Because of the similarity in structure between nicotine and compounds that have been shown to induce PD, we hypothesized an interaction between nicotine and synthetic NM-Fe and later reported that nicotine binds to neuromelanin in a saturable manner, with a K_d_ value of 0.65 mM [23]. Our hope is that this single assay may provide utility in competition assays used for the measurement of binding constants of numerous other chemical agents and is the driving force behind for the current report.

### 1.3. Hypothesis of Role for NM in Binding Dopamine

Beyond this, we reasoned that if neuromelanin is functioning in the cell as a toxin-binding agent, there must be an endogenous ligand as well that is normally present in the cell. We hypothesized that dopamine is an obvious potential ligand, and the regulation of dopamine molecules in the cell for uptake and release has been proposed as a simple memory system involving neuromelanin granules [23]. The ability to bind and retain monomeric forms of macromolecular polymer is a critical yet often overlooked self-reinforcing mechanism for the growth of living organisms. Notably, polymeric forms of the nucleic acids RNA and DNA attract free nucleotides [24], peptides attract free amino acids, and glycogen and similar sugar polymers attract free sugars, if only through a like-dissolves-like function of intermolecular forces. Before the existence of transporters, receptors, and concentrating hormones, there were only the macromolecules themselves to support these functions. We have hypothesized that neuromelanin also falls into this category, that it has a primary function in binding, storage, and maintenance of cellular dopamine concentrations, and that this is a critical yet largely unappreciated phenomenon [25]. As noted, neuromelanin is well known for binding iron. In doing so, it becomes a very real attractive force for lone electron pairs such as those of heterocyclic nitrogen-containing aromatic rings or aromatic phenyl rings with an attached monoamine as in the case of dopamine and epinephrine.

There are several potential obvious advantages to such an attraction and accumulation mechanism. Perhaps most interesting among these is that dopamine oxidation to the quinone releases electrons. While stressful in terms of cell oxidation state, this also provides a potential source of energy. It is entirely plausible that dopamine polymerization and subsequent quinone formation provides electron flow within the inner membrane of the double-membrane-bound NM granule, reminiscent of the role of ubiquinone employed during mitochondrial electron transfer. We are inclined to investigate the possibility that the NM granule is a remnant of an early energy generation system, working in concert with mitochondria to provide electrical gradient energy. Very recently, it was reported that the NM granule may act to supplement the action of lysosomal degradative pathways when such pathways are otherwise impaired [26], again highlighting the attractive nature of the granule itself.

### 1.4. Summary

In our previous work, we noted the discovery of a fluorescence signal induced by nicotine in the presence of NM. The present study was begun with two goals in mind. Firstly, we hoped to demonstrate the general utility of the nicotine/NM competition assay for the estimation of binding affinities to NM of difficult-to-measure compounds such as dopamine. Toward this goal, we investigated sources of error and variability in our experimental protocol, including long-term solution storage and the role of the oxidation state of the bound iron atoms to the fluorescence signal of NM/nicotine. Secondly, we asked whether 6-hydroxydopamine will also polymerize into neuromelanin in vivo and if so, what the consequences of this are in comparison to more natural melanin. Unlike most biological polymers, melanins do not depolymerize through simple hydrolysis, such that incorporation of odd entities would be a permanent proposition. We have found that synthetic NM ‘spiked’ with 5% of the normal dopamine replaced by 6-hydroxydopamine has unique properties compared to ‘normal’ NM, inasmuch as it exhibits a much higher propensity to form a hydrogel state (unpublished observations).

## 2. Materials and Methods

### 2.1. Synthesis of Model Neuromelanin

Synthetic neuromelanin was prepared as adapted from others [5] and described previously [23]. This polymer was used in all analytical procedures in the current work unless otherwise noted. First, 0.33 mmol (58 mg) L-cysteine hydrochloride monohydrate and 2.0 mmol (370 mg) dopamine hydrochloride were dissolved in 200 mL of phosphate buffered saline (PBS) in a brown glass bottle. This was placed in a 37 °C incubator and allowed to autooxidize open to air for approximately 120 h. The solution was then placed into microcentrifuge tubes to allow for centrifugation at high speed. The tubes were subjected to centrifugation at 10,000 RPM for 10 min in order to separate the heavier polymer from the excess L-dopa. The supernatant was discarded, and the pellet was resuspended with 0.33 mmol cysteine in 200 mL PBS. This mix was autooxidized at 37 °C for 48 h and then subjected to low-speed centrifugation. The supernatant was discarded, and the pellets were resuspended in 200 μL of 1% acetic acid. The tubes were again subjected to centrifugation and the supernatant was discarded.

To chelate any residual metals, the solutions were each resuspended twice with 100 μL of 0.15 M disodium EDTA and twice with 500 μL distilled deionized water and the centrifugation repeated to remove the supernatant after each resuspension. The pellets were recombined and resuspended with 2 mL of water. The slurry was then pipetted into autoclaved dialysis tubing (12,000–14,000 molecular weight cutoff) treated with EDTA and dialyzed with gentle stirring in 2 L of distilled deionized water for 120 h; the dialysis water was replaced once. Approximately 9.1 mL of the resulting purified NM was reacted with 0.769 mL of 1.3 M iron(III) chloride (FeCl_3_) in order to sequester iron(III) ions within the polymer to form NM-Fe^3+^ or activated NM. This was finally subjected to centrifugation in order to pellet the unreacted NM, which was discarded. The activated NM-Fe^3+^ supernatant was removed and dialyzed against water without stirring for approximately 168 h to remove unbound ions.

### 2.2. Selection of Materials

A NanoDrop 3300 Fluorospectrometer (Thermo Fisher Scientific, Waltham, MA, USA) was used for all fluorescence measurements. This fluorimeter is only capable of illuminating the sample in 3 ‘blocks’ of filtered visible light, rather than a full spectrum as is traditionally performed by a diode array or prismatic system of a larger instrument. A larger instrument however would also require cuvettes, something that is prohibitive to performing experiments with limited quantities of neuromelanin, while the NanoDrop requires only a sample size of 1–2 µL. As such, a 2 µL aliquot of each mixture was placed onto the instrument pedestal. Excitation with the white visible light filtered source followed by emission Relative Fluorescence Units (RFU) measured at 530 nM was found to be indicative of nicotine interactions with NM and was used for all measurements in the current study.

Nicotine (Nic) samples were prepared fresh and discarded after about a month of use due to inconstancies of unknown origin noted with older diluted samples. A variety of samples of NM that had been prepared and saturated with iron according to published procedures [5,23] were initially sampled. A dilution series of samples were prepared both with distilled water and separately with nicotine. A sample of iron-treated NM was selected for use in all following tests by having the highest relative increase in fluorescence when diluted with nicotine, implying the largest increase in NM-Nic complex concentration. Later experimental evidence with sodium dithionite suggested that the iron needs to be in the Fe^2+^ state, such that iron added in the Fe^3+^ state during these experiments had presumably been reduced by residual dopamine oxidation.

A series of NM samples were prepared of varying dilution factor with both water and nicotine as the diluent such that in nicotine-treated samples the nicotine was in clear excess. The relative increase in fluorescence was noted for each sample, and a dilution factor of 5 was chosen for the remainder of the assay development, on the grounds that the increase in fluorescence was significantly high at this level, but that the fluorescence of the untreated NM was visible as well. Therefore, the progression of the reaction may then be monitored. This exact dilution factor is not entirely necessary for the assay, but it is the value with which the methods were developed.

### 2.3. Nicotine Fluorescence Binding Assay

Samples of MPP+, dopamine, and 6-hydroxydopamine were prepared at a concentration of approximately 1.625 mM. A serial dilution was performed to prepare small samples of each analyte at every order of magnitude down to the femtomolar level using deionized water as the diluent. For each analyte, a control trial was performed using 4 µL of sample, 4 µL of deionized water, and 2 µL of neuromelanin. The test sample was vortexed briefly and their fluorescence measured at a wavelength of 530 nm. This control proves that the theoretical complex of neuromelanin with the analyte does not fluoresce significantly on its own. Following the control, an experimental competition-binding assay was performed for each analyte using 4 µL of sample, 4 µL of 1.625 mM nicotine, and 2 µL of neuromelanin. Just as with the control, the test samples were vortexed briefly and fluorescence was measured at 530 nm. The expected trend of this trial was that of a dose-response curve, and the program GraphPad Prism^TM^ (Version 8, GraphPad Software, San Diego, CA, USA) was used to monitor the resultant outputs, specifically the IC_50_ value of the analytes with neuromelanin. All data points shown in the current work represent an average of quadruplicate measurements. 

### 2.4. Nicotine Fluorescence Assays Performed under Reducing Conditions

NM/Nicotine fluorescence was assayed as above using a NanoDrop 3300 (Thermo Fisher Scientific, Waltham, MA, USA) with white light excitation. The concentration of nicotine was varied from 2.0 mM and below, depending on the assay. Solutions of NM, nicotine, and sodium dithionite (or water for control experiments) were added in a 1:1:1 ratio. A 1:250 dilution of 0.092 g per 1.0 mL H_2_O Sodium Dithionite (Na_2_S_2_0_4_; 0.528 mM) was chosen for all the collection of experimental data. Excitation Fluorescence results in RFU were recorded and graphed using Microsoft Excel^TM^.

### 2.5. Cell Culture and Immunohistochemistry

Rat substantia nigra neurons (Cat. #R1550) were purchased from ScienCell Research Laboratories^TM^ (Carlsbad, CA, USA) along with neuronal growth medium, antibiotic/antifungal mix, and differentiation growth factor. These cells were plated and grown in 4-well, treated chamber slides for approximately 3–4 weeks, after which time L-DOPA and or 6-OH L-DOPA (2,4,5-trihydroxy-l-phenylalanine) were added to final concentrations of 50 µM (control contained only the phosphate buffered saline solution used to dilute test compounds). Treated media was replaced on alternating days. After 9 days of treatment the interior of the cells as well as the external media became very dark at which time the spiked media were replaced with untreated, fresh media and cells grown for approximately 12 more days to allow for recovery and melanin granule formation prior to analysis. 

For visualization of bound dopamine, immunohistochemical analysis was carried out using standard protocols. All steps were carried out at room temperature. Cells were washed with PBS (3 × 5 min), fixed with 4% paraformaldehyde (15 min), and washed with PBS/0.1% TWEEN^R^ 20 (3 × 5 min) prior to incubation with antibody. The primary antibody used was a polyclonal preparation raised in rabbit against dopamine conjugated to a larger carrier molecule (Abcam Cat#ab6427; Cambridge, MA, USA). It was diluted 1:200 in PBS/Tween and incubated for 30–60 min. Goat anti-rabbit secondary antibody labeled with a green fluor tag (Alexa Fluor^TM^ 488; Abcam, Cambridge, MA, USA) was used at a 1:1000 dilution and incubated for 30–60 min. Images were collected using an EVOS M5000 benchtop inverted fluorescence microscope (Thermo Fisher Scientific, Hanover Park, IL, USA)

## 3. Results and Discussion

### 3.1. General Utility of Nicotine as a Signal Molecule for Measurement of Competition-Binding to Synthetic Neuromelanin

The measurement of binding affinity of compounds to neuromelanin by traditional spectroscopic means is problematic for several reasons, primary among them the strong and very broad absorbance of the melanin itself. Our longstanding interest in the measurement of the binding affinity of dopamine to synthetic NM presents additional problems in that the NM polymer is derived from that very dopamine monomer, such that the dopamine being tested can conceivably add to the growing polymer rather than displaying equilibrium binding kinetics. The use of a signal molecule such as nicotine avoids this problem, such that nicotine should have general utility in the determination of the binding affinity for many test compounds.

The fluorimeter was blanked with water followed by test solutions formed from 1:1:1 volumetric ratios of synthetic NM, 1.95 mM nicotine (or water, for control measurements), and test compound. The final nicotine concentration is thus maintained near its measured K_d_ of 0.65 mM, thereby simplifying and enabling the estimate of competitor K_d_, while the concentration of competitor molecule is varied. In the current study, we chose three model compounds for testing with our competition assay based on their central role in the understanding of Parkinson’s disease. These compounds were 1-methyl-4-phenylpyridinium ion (MPP+) and 6-hydroxydopamine, both potent initiators of Parkinson’s symptoms in animal models, and dopamine itself, the neurotransmitter that becomes in short supply during latter stages of the disease.

**Figure 1:**
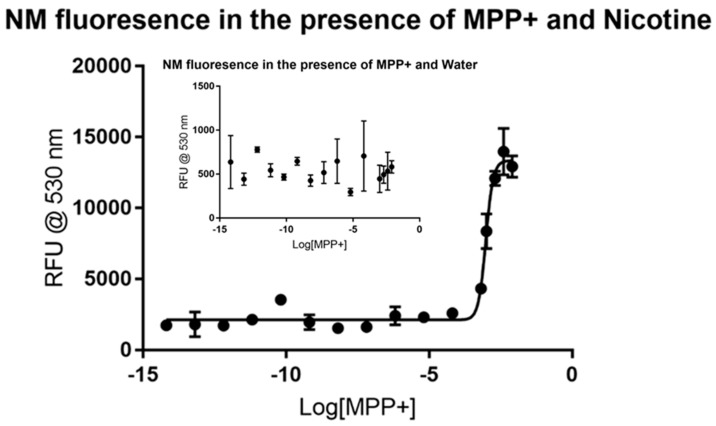
Fluorescence of synthetic neuromelanin (NM; sometimes written as NM-Fe to indicate its iron-bound state) upon exposure to 1-methyl-4-phenylpyridinium ion (MPP+) in the absence (inset) and presence of nicotine as a signal molecule. MPP+ does not itself appear to induce a stable fluor signal in solutions of NM-Fe as judged by the high variability and low average RFU counts of quadruplicate measurements (inset). On the inclusion of nicotine however, an enhanced signal appears to stabilize slightly, with a sharp inflection evident near 10^−3^ M MPP+ concentrations (K_d_ ~1 mM). A second, smaller inflection was consistently evident in repeat experiments as seen by the hump near 10^−11^ M, suggestive of a second saturable binding interaction at much lower concentrations.

The potent Parkinsonian-inducing agent 1-methyl-4-phenylpyridinium ion (MPP+) is known to exert its toxic effects on the mitochondria of dopaminergic neurons following its uniquely strong sequestration into these cells. The accumulation into dopaminergic neurons is thought to be a combination of transporter action (dopamine transporters/DATs and vesicular monoamine transporters/VMATs) and binding to neuromelanin [15,16,17]. Nicotine has been shown to protect against MPP+ toxicity both in human epidemiological studies as well as animal models [19,27]. The control analysis of MPP+ and neuromelanin showed no major spike in fluorescence due to increasing the concentration of MPP+, as illustrated in the inset portion of Figure 1. A possible inflection emerges but no viable trend can be observed. Upon the addition of nicotine as a signal molecule, however, the resulting data spikes at the millimolar level, as shown in the main figure. Non-linear regression analysis of the dose-response curve suggests that MPP^+^ has an EC_50_ (1/2 maximal effective concentration) between 0.8883 and 1.052 mM with 95% confidence. The dose-response curve fits to the data with an *R*^2^ value of 0.9685. An additional interesting spike in RFU appears near 10^−10^ M MPP+, suggesting a possible saturation point at very low concentrations. This signal appears to be then rapidly quenched by the addition of further MPP+, followed by the second, more clearly visible saturable increase in RFU that appears 7 or 8 orders of magnitude above the first. The fact that the application of MPP+ appears acts as an agonist to the formation of the fluorescing NM-Nic complex is surprising. All analytes were expected to act as antagonists to NM-Nic formation. The mechanism by which MPP+ is driving neuromelanin to bind nicotine is unknown, but its application certainly is altering the normal binding behaviors of neuromelanin.

**Figure 2:**
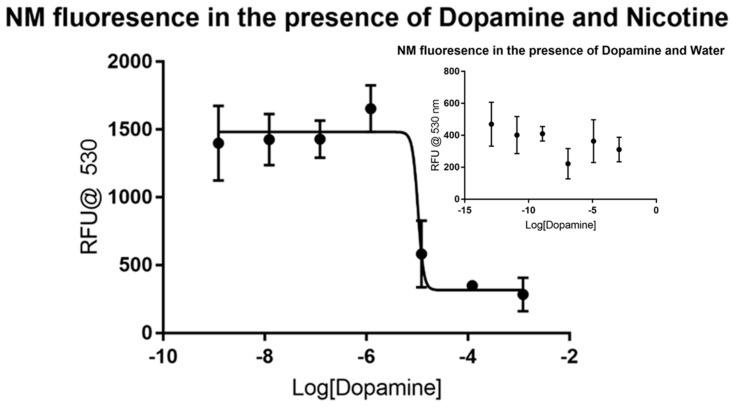
Fluorescence of synthetic neuromelanin upon exposure to dopamine in the absence (inset) and presence of nicotine as a signal molecule. Like the situation with MPP+ from Figure 1, dopamine does not itself appear to induce a stable fluor signal from NM-Fe in aqueous solution as judged by the high variability and low average RFU counts of quadruplicate measurements (inset). On the addition of nicotine, an enhanced signal appears to stabilize slightly with a sharp inflection evident near 10^−5^ M dopamine, in agreement with our prior published estimate of an 0.05 mM K_d_ [23].

**Figure 3:**
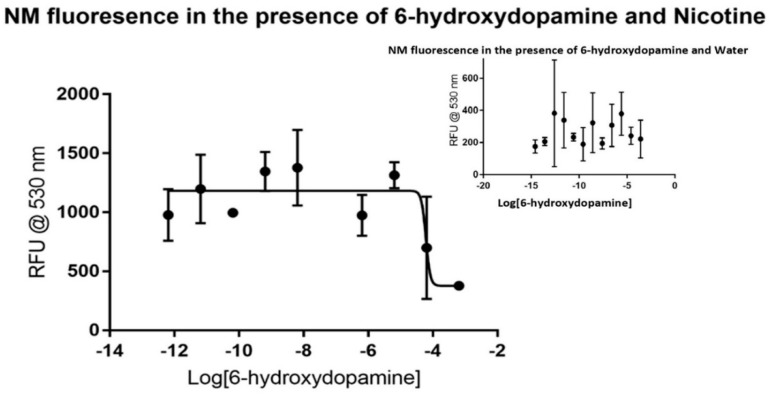
Fluorescence of synthetic neuromelanin upon exposure to 6-hydroxydopamine in the absence (inset) and presence of nicotine as a signal molecule. As with MPP+ and dopamine, 6-hydroxydopamine itself did not appear to reveal a signal that could be related to saturation binding kinetics when added to NM, but in the presence of nicotine displayed increased and more stable average quadruplicate RFU values. In this case, an inflection appears near 10^−4^ M 6-hydroxydopamine by non-linear regression analysis, suggestive of a saturable binding affinity in the 0.1 mM range for 6-hydroxydopamine to NM-Fe^3+^ in buffered aqueous solution. As in all prior cases, points presented are the average of quadruplicate measurements with error analysis performed with GraphPad Prism^TM^.

Fewer data points were gathered for dopamine and 6-hydroxydopamine considering the rapid degradation of the samples that occur without additional and costly precautions. Dopamine did not produce a visible trend in the control, as shown in the inset portion of Figure 2, making it a viable candidate for the assay. The dose-response curve produced by using nicotine showed major inhibition at the micromolar level, as shown in the larger plot. Non-linear regression analysis reveals that dopamine has an IC_50_ value of around 10.44 µM without a defined confidence interval under these conditions. The dose-response curve generated has an *R*^2^ value of 0.9077 and 23 degrees of freedom. Like dopamine, 6-hydroxydopamine did not produce a visible trend in the control, as shown in Figure 3. The dose-response curve produced by using nicotine showed major inhibition at the micromolar level, but with less certainty than that of dopamine. Non-linear regression analysis shows that 6-hydroxydopamine has an IC_50_ value of around 61.03 µM without a defined confidence interval. The dose-response curve generated has an *R*^2^ value of only 0.5572 with 30 degrees of freedom.

### 3.2. Redox State of Iron-Bound to Synthetic NM

In working with synthetic NM samples and multiple users over a period in our laboratory, it was noted that while the fluorescence signal was stable and reproducible for a period of weeks, it did not appear to be stable over the longer term (months). Various storage methods involving brown glass bottles or small sealed plastic tubes of polymer kept in a dark cupboard were employed during this time as we had anticipated possible effects of exposure to atmospheric oxygen and light from the laboratory fixtures and windows on the highly pi-conjugated system of NM. We presumed that polymerization had ceased following our synthesis procedure. We were confident that the iron that had been added remained bound owing to the consistent color of our samples which revealed no evidence of iron leaching either out of our polymer nor into our test solutions after dialysis, storage, and usage and presumed that this iron had reached an air-oxidized Fe^3+^ state on the order of days.

Neuromelanin isolated from humans exhibits a relatively low-affinity, reduced catechol binding site as well as a higher-affinity site for oxidized quinone forms that exist in a catecholamine redox equilibrium on the path toward polymerization of the oxidized forms [28]. Because modeled NM-Fe displays characteristics resembling a sheet of heme moieties that have been sewn together into a giant quilt-like pattern [29,30,31,32], and in part because of our troubles with aging NM samples, we decided to test the reduction state of Fe bound to NM by adding a reducing agent that would ensure all of the bound Fe was at least temporarily in the Fe^2+^ state, rather than the Fe^3+^ state that we had been assuming our measurements were made under. Sodium dithionite, a reagent commonly used for the quantification of cytochrome P450 heme-containing enzymes (absorbance at 450 nm in the oxidized vs reduced state in the presence of carbon monoxide is what gives the characteristic P450 superfamily their name), was chosen for this purpose. Research into the use of sodium dithionite for a bleaching step during leather tanning indicated that a 1% dosage of dithionite combined with sodium sulfite was enough to remove melanin during enzymatic dehairing of calf skin [33].

**Figure 4:**
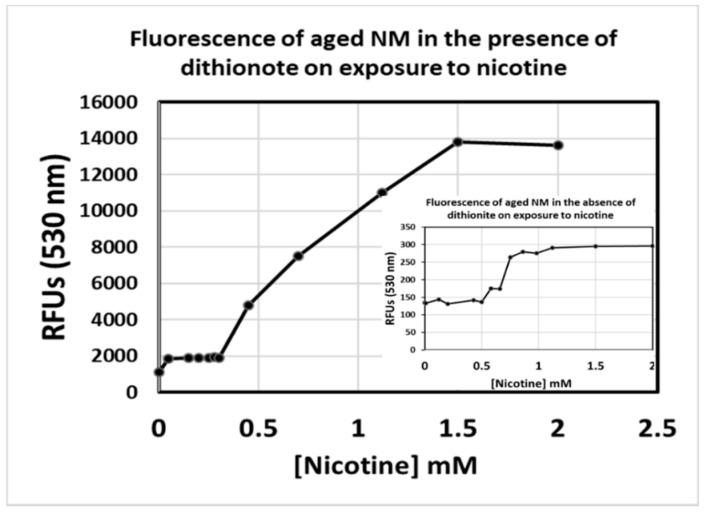
Fluorescence of synthetic neuromelanin upon exposure to nicotine in the absence (inset) and presence of sodium dithionite as a reducing agent. Synthetic NM-Fe^3+^ that had been aliquoted into a sealed, 15 mL conical tube and stored in a dark cupboard for over 3 months was tested for signal upon addition of nicotine as per a slight variation on our published procedure (emission monitored at a wavelength of 530 nm; inset). The nicotine still appears to saturate in the 0.65 mM range, but RFU values are some 2-orders of magnitude lower than expected (inset). Upon addition of 1% sodium dithionite, signals matching the rang of RFU values seen when the batch of NM-Fe was first synthesized were instantly restored. Values presented are the average of triplicate measurements.

When added to our synthetic NM, the dithionite caused an evident visual bleaching. Samples tested at a maximum dithionite solubility of 18.2 g/100 mL (0.182 g/1 mL) were used in first assays but were found unreadable by the NanoDrop 3300 due to high RFU values. The sodium dithionite solution was eventually diluted to 1:250 from its original saturated state, at which point some visual bleaching was still evident upon addition to neuromelanin. RFU data are shown in Figure 4, where the values for nicotine/NM collected from aged polymer (>6 months storage in the dark) in the absence of added dithionite were very low (inset). Notably however, we still observe an inflection point near the same concentration of nicotine as that collected from fresh sample. Under the reducing conditions of dithionite inclusion, much higher RFUs for the nicotine/NM interaction appear, a result that was quite to our surprise. It suggests that rather than remaining in the Fe^3+^ state after iron addition, dialysis, and exposure to air, our synthetic NM-Fe instead appears to quickly reach and remain in the reduced Fe^2+^ state for a period of weeks, and that this Fe^2+^ state is necessary for the observation of the fluorescence signal during the nicotine saturation assay. This agrees with more recent reports revealing that the NM polymer retains a significant amount of unpolymerized, caged dopamine monomer, and that it continues to polymerize slowly over a period of weeks [34,35,36]. In doing so, polymerization will necessarily release electrons into the pi-conjugated system due to the nature of the catechol/quinone redox process, and, it seems, will maintain bound iron in the reduced Fe^2+^ state as well during this time.

### 3.3. Polymerization and Incorporation of Dopamine, 6-Hydroxydopamine, or Combinations of Both into Neuromelanin Granules in Cultured Neurons

Given our hypotheses and developing theory surrounding the function of the neuromelanin granule in the storage and release of dopamine, we noted that this hypothesis also provides a possible explanation and a target for the toxic effects of 6-hydroxydopamine, something that to our knowledge has not been established in the literature. In particular, we have long suspected that 6-hydroxydopamine could exert its toxicity by binding to an existing NM granule in place of dopamine, hypothesizing that the NM functions to bind, store, and release dopamine, and that due to similarity in structure but higher reactivity toward quinone formation, the presence of 6-hydroxydopamine would serve to prevent dopamine binding and storage by binding in its place and/or by cross-linking the interior of the NM granule. Alternatively, 6-hydroxydopamine could form abnormal melanin polymers that were immediately toxic to cells unfortunate to encounter them. As such, we were eager to test dopaminergic neurons that had been differentiated in culture from commercially available rat substantia nigra tissue for their ability to take up and possibly polymerize 6-hydroxydopamine. To achieve this, we used the published procedure for the creation of pigmented neurons but substituted the zwitterion precursor of 6-hydroxydopamine, known as 2,4,5-trihydroxy-l-phenylalanine, in place of levodopa. We anticipated that under these circumstances, a large portion of the added 2,4,5-trihydroxy-l-phenylalanine would be rapidly decarboxylated through the action of dopa-decarboxylase, analogous to the conversion of levodopa to dopamine.

**Figure 5:**
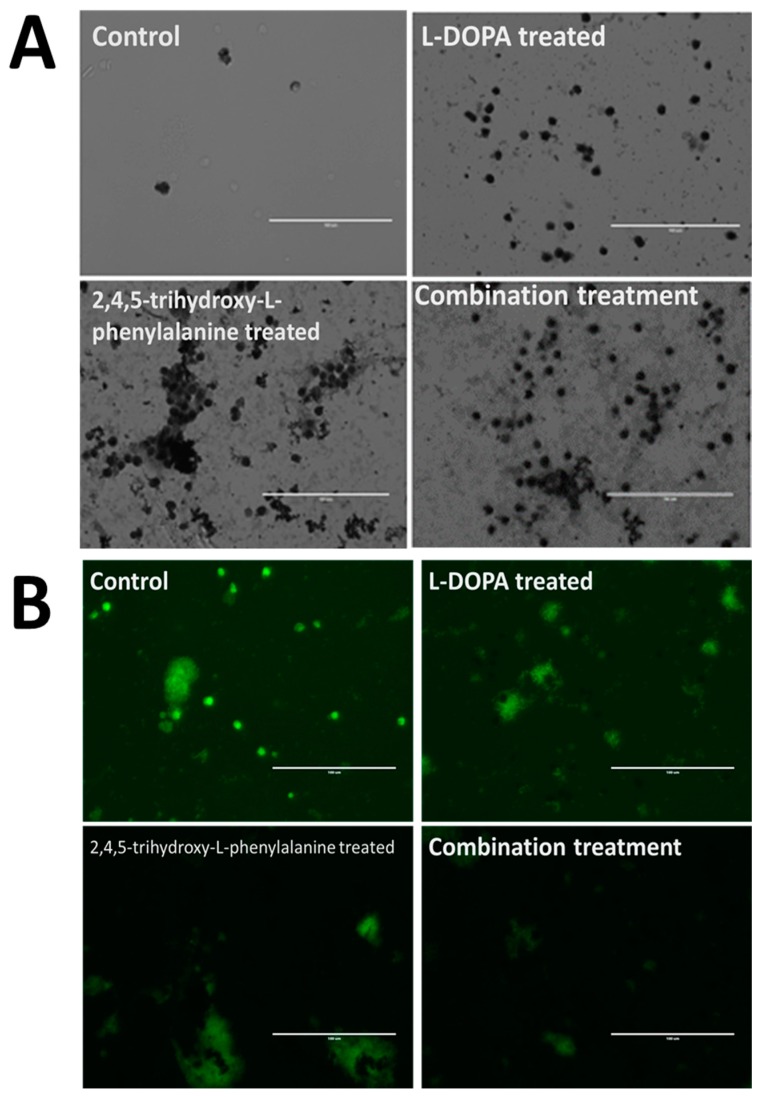
Incorporation of catecholamine polymers into neuromelanin granules following exposure to precursor molecules in cultured neurons. Rat fetal Substantia nigra tissue was purchased commercially and induced to form dopaminergic neural networks according to published procedures [5]. Addition of control buffer solution (panels A and B, upper left frame) is shown compared with additions of 50 uM levodopa (l-DOPA; l-3,4-dihydroxyphenylalanine; Panels A and B; upper right frame) or 50 uM 6-hydroxy-levodopa (6-hydroxy l-DOPA; 2,4,5-trihydroxy-l-phenylalanine; Panels A and B; lower left frame) and a combination of both (panels A and B; lower right frame). Images were taken using an EVOS tabletop fluorescence microscope under visible light conditions (Panel A) or using the appropriate fluor filter (Panel B). Control cells are faint and difficult to see under these conditions due to the lack of accumulated melanin. Levodopa, which is presumably decarboxylated rapidly to dopamine upon entering the cell through the membrane as a neutral zwitterion, caused the cells to display an initial shocked period during catecholamine polymerization and accumulation into NM granules, recovering to a healthy state over a period of weeks. The 6-hydroxydopamine counterpart to levodopa (2,4,5-trihydroxy-l-phenylalanine) displayed similar characteristics but resulted in a noticeably more darkened pigmented granule. Dopamine appears less localized in cells that have accumulated melanin, with this effect being most pronounced in those cells treated with a combination of l-DOPA along with the 6-hydroxy counterpart.

Figure 5 shows the results of such exposure after giving the cells a period of 1–2 weeks without added catecholamine in order to recover from the redox-shock. As seen in the upper left panel, control cells are faint and difficult to see under these conditions due to the lack of accumulated melanin. Levodopa (upper right corner of panel A) which is presumably decarboxylated rapidly to dopamine upon entering the cell through the membrane as a neutral zwitterion, caused the cells to display an initially unhealthy appearance during catecholamine polymerization and accumulation into NM granule stages, but these cells recovered to a healthy state over a period of days/weeks. The 6-hydroxydopamine counterpart to levodopa (2,4,5-trihydroxy-l-phenylalanine) displayed similar characteristics but resulted in a noticeably more darkened pigmented granule. A combination of 50:50 resulted in granules that are intermediate in darkened-appearance. These results suggest that 6-hydroxydopamine is capable of substituting for dopamine in a neuromelanin polymerization sequence and appears further able to be packaged into stable granules. It is unknown at this time what other functional effects this polymer may have imparted to the cell, though we hypothesize that it would be distinct in activity from the normal dopamine polymer due to the added hydroxyl group. In vitro experiments with synthetic NM made using 6-hydroxydopamine show that the altered polymer exhibits unusual characteristics, forming a hydrogel-like state in aqueous solutions. While ‘normal’ NM will also form this hydrogel, it will only do so following exposure to salt, while the 6-hydroxydopamine altered form does this in water alone (unpublished result).

## 4. Conclusions

The nicotine/NM competition fluorescence assay appears to be viable and highly useful for the estimation of binding constants toward NM of a variety of compounds that may themselves not elicit a signal. The inhibitory constants of dopamine and 6-hydroxydopamine measured in the current study are at a level consistent with concentrations expected to be found in the cell. The micromolar level is the higher end of this threshold so while it is not impossible that dopamine or dopamine derivatives are an endogenous ligand, other possibilities for neuromelanin binding cannot be ruled out.

This experiment is only able to reach limited conclusions regarding dopamine and 6-hydroxydopamine binding behaviors over short time periods due to the molecules’ nonideal behavior in aqueous environments. That is, in our limited in vitro assay system, higher concentrations of catecholamine discolor quickly, signifying oxidation and polymerization, or otherwise degrading or eliminating processes, making the samples more difficult to measure with confidence at higher range over extended time periods. We have yet to include lipid and protein normally associated with neuromelanin granules, nor have be attempted to created synthetic membrane-bound forms to work with that better mimic the natural state. Additionally, the spectrofluorometer used was at times inconsistent in measuring the fluorophores present. This may be due to a lack of understanding of the NM-Nic complex itself and what conditions best amplify the signal, unexpected aggregation upon ligand binding, or other phenomena yet to be elucidated.

One of the biggest issues discovered in the procedure of this experiment that required controlling for is an apparent time dependency of nicotine used as a signal molecule. Older samples of nicotine produced fluorescence results inconsistent with those produced using freshly prepared nicotine. The mechanism by which the change happens is unknown and further study may reveal the optimal conditions for the use of nicotine as a signal, or perhaps research will find an alternative signal molecule that does not degrade over time. However, this issue is easily avoided by preparing nicotine dilutions freshly for assay work in small batches so long as this phenomenon is kept in mind. In addition, we have found that fresh synthetic NM most likely exists in the Fe^2+^ reduced state, and that eventual air oxidation to the Fe^3+^ -state prevents or dampens the RFU signal considerably. We have shown that reduction back to the reduced state appears to fully restore signal. Finally, this assay could be greatly improved with the addition of a higher throughput fluorescence detector. Running one sample at a time is highly inconvenient, especially regarding samples such as dopamine and 6-hydroxydopamine which would benefit from rapid detection of all samples. 

## Abbreviations

K_d_	Dissociation constant
NM	Neuromelanin
NM-Fe^3+^	iron-treated neuromelanin
PBS	phosphate buffered saline
RFU	relative fluorescence units
PD	Parkinson’s disease
UV/Vis or UV/visible spectroscopy	ultraviolet/visible spectroscopy
